# Malignant obstruction of the inferior vena cava: clinical experience with the self-expanding Sinus-XL stent system

**DOI:** 10.1007/s00261-022-03587-1

**Published:** 2022-07-06

**Authors:** Anne Marie Augustin, Leonie Johanna Lucius, Annette Thurner, Ralph Kickuth

**Affiliations:** Department of Diagnostic and Interventional Radiology, University Hospital of Würzburg, Oberdürrbacher Strasse 6, DE 97080 Würzburg, Germany

**Keywords:** Endovascular, Inferior vena cava, Interventional procedures, Oncology, Palliative care, Stent

## Abstract

**Purpose:**

To evaluate the technical and clinical outcome of Sinus-XL stent placement in patients with malignant obstruction syndrome of the inferior vena cava.

**Methods:**

Between October 2010 and January 2021, 21 patients with different malignant primary disease causing inferior vena cava obstruction were treated with Sinus-XL stent implantation. Procedural data, technical and clinical outcome parameters were retrospectively analyzed.

**Results:**

Technical success was 100%. Analysis of available manometry data revealed a significant reduction of the mean translesional pressure gradient following the procedure (*p* = 0.008). Reintervention rate was 4.8% (1/21). The available follow-up imaging studies showed primary and primary-assisted stent patency rates of 93% (13/14) and 100% (14/14), respectively. Major complications did not occur. The clinical success regarding lower extremity edema was 82.4% (14/17) for the first and 85.7% (18/21) for the last follow-up. Longer lengths of IVC obstruction were associated with reduced clinical improvement after the procedure (*p* = 0.025). Improvement of intraprocedural manometry results and lower extremity edema revealed only minor correlation. Ascites and anasarca were not significantly positively affected by the procedure.

**Conclusion:**

Sinus-XL stent placement in patients with malignant inferior vena cava obstruction showed high technical success and low complication rates. Regarding the clinical outcome, significant symptom improvement could be achieved in lower extremity edema, whereas ascites and anasarca lacked satisfying symptom relief. Based on our results, this procedure should be considered as a suitable therapy in a palliative care setting for patients with advanced malignant disease.

## Introduction

In malignant inferior vena cava (IVC) obstruction, advanced primary malignancy or metastatic disease of organs surrounding the IVC lead to IVC compression, invasion or thrombus formation with impaired venous return to the heart [1]. Compared to malignant obstructions of the superior vena cava (SVC), IVC compression-syndrome is rare [2].

Symptoms of malignant IVC obstruction vary, depending on degree and level of obstruction, time of onset and duration of vessel compression [3, 4]. Patients may suffer from hypotension, tachycardia, lower extremity edema, anasarca, skin ulcerations and eczema, ascites as well as renal and hepatic insufficiency. Thus, in severe cases the quality of life and the capability of undergoing therapy may be considerably limited [5, 6].

Since symptoms of malignant infra-diaphragmatic IVC obstructions use to occur late in the course of an advanced malignant disease, therapy concepts are predominantly palliative [7]. These vulnerable patients are poor surgical candidates and non-invasive, medical therapies such as diuretics lack the desired efficacy. Hence, additional treatment strategies are required to palliate these patients sufficiently [8].

Percutaneous endovascular treatment of IVC obstructions with stent placement represents a minimally invasive alternative in terms of prompt hemodynamic therapy effect and fast symptom relief [9]. To date, only few data exist addressing the technical success of IVC stent placement, and even less regarding the clinical outcome of the treated patients. Therefore, the purpose of this study was to evaluate our clinical experiences using the self-expanding Sinus-XL stent system (Optimed, Ettlingen, Germany) for treatment of patients with symptomatic IVC obstruction due to malignancies of various origin.

## Materials and methods

### Study cohort

A retrospective review of the archives of our interventional radiology department from October 2010 to January 2021 yielded the cases of 21 patients with malignant IVC obstructions (11 women and 10 men, median age 61.0; range 18–92 years), who underwent IVC stent implantation. In each case, we used the closed-cell designed, self-expanding nitinol Sinus-XL stent. Patients with benign IVC compression were excluded from the study. The requirement for consent from patients to be included in this retrospective study was waived by our institutional review board (No. 20211125 01). All patients were examined and treated as part of routine care and informed consent for the procedure was obtained before the procedure. Patients’ characteristics and underlying conditions are presented in Table 1.

**Table 1:** Patients’ characteristics

Patient	Age	Gender	Primary diagnosis	Cause of obstruction	Level of obstruction	Previous treatment	Subsequent treatment
1	91	F	MM	Primary tumor	Suprarenal	RCTX	–
2	42	F	ACC	Hepatic and lymphonodal metastasis	Intrahepatic to renal	CTX	–
3	44	M	NSCLC	Hepatic and lymphonodal metastasis	Intrahepatic and renal	RCTX	–
4	35	F	B-ALL	Lymphonodal	Supra- and infrarenal	CTX	RCTX
5	18	M	Testicular cancer	Lymphonodal	Renal to iliac	CTX	CTX
6	64	F	ACC	Primary tumor	Intrahepatic to iliac	CTX	CTX
7	72	M	NHL	Lymphonodal	Supra- and infrarenal	CTX	CTX
8	61	F	NET Pancreas	Hepatic metastasis	Intrahepatic	RCTX	CTX
9	61	M	NET Ileum	Hepatic metastasis	Intrahepatic	CTX	–
10	53	F	Anal cancer	Hepatic metastasis	Intrahepatic	RCTX	–
11	25	F	Leimyosarcoma	Hepatic metastasis	Intrahepatic	RCTX	CTX
12	55	F	CCC	Primary tumor	Intrahepatic	RCTX	RCTX
13	72	F	AEG II/III	Lymphonodal	Intrahepatic	RCTX	–
14	79	F	HCC	hepatic metastasis	Intrahepatic	CTX	CTX
15	44	M	SCLC	Hepatic and lymphonodal metastasis	Intrahepatic and infrarenal	RCTX	–
16	63	M	HCC	Primary tumor	Intrahepatic	–	–
17	57	F	NET unknown origin	Lymphonodal	Supra- and infarenal	RCTX	RTX
18	79	M	NET Ileum	Lymphonodal	Infrarenal to iliac	RCTX	–
19	63	M	Liposarcoma	Primary tumor	Infrarenal to iliac	RCTX	CTX
20	92	M	Gastric cancer	Hepatic metastasis	Intrahepatic to renal	–	–
21	69	M	CUP	Lymphonodal	Infrarenal	CTX	CTX

*ACC adrenocortical carcinoma, B-ALL B-cell acute lymphoblastic lymphoma, CTX chemotherapy, CUP cancer of unknown primary, MM multiple myeloma, NET neuroendocrine tumour, NHL non-Hodgkin´s lymphoma, NSCLC non-small cell lung cancer, RTX radiotherapy, RCTX radio-chemotherapy*

IVC obstruction syndrome was based on primary tumor compression in five cases, whereas in 16 cases, significant IVC compression was caused by metastatic disease, including metastatic lymph nodes in seven, hepatic metastases in six and a combination of lymph node and hepatic metastases in three patients.

In each case, the indication for endovascular IVC stent implantation was approved by an interdisciplinary team consisting of interventional radiologists, oncologists as well as visceral and vascular surgeons. In 20 patients, stent placement was triggered by worsening of IVC obstruction symptoms. In one case, stent placement was performed as a preventive measure.

### Preprocedural imaging

For verification of IVC obstruction and procedure planning, 19 patients (90.5%) underwent abdominal contrast-enhanced computed tomography (CT), one patient received abdominal contrast-enhanced magnetic resonance imaging (MRI), and one patient was examined by ultrasound. Diagnostic imaging was performed at a median of 8 days (range 0–64 days) prior to the procedure.

### Procedure

All procedures were performed by the same operator in our local angiography suite (Siemens, Axiom Artis Zee, Forchheim, Germany) via a transfemoral antegrade venous approach. In 20 cases, we used the right common femoral vein as access site. In one case, the left common femoral vein was accessed. IVC stenosis was passed with a 0.035-inch guide wire (Radifocus, Terumo, Tokyo) and a 5F selective angiography catheter (Berenstein configuration, Angiodynamics, Queensbury, NY). IVC stenosis was confirmed by digital subtraction cavography via a 10F sheath (Check-Flo, Cook, Bjaeverskov, Denmark). Prior to cavography, we exchanged the crossing guide wire and the selective catheter for a 0.035-inch support guide wire (Back-Up Meier, Boston Scientific, Natick, Mass) and a calibrated 5F pigtail catheter. We chose oversized self-expanding stents exceeding the normal IVC diameter by approximately 25–30%. The Sinus-XL stent is a laser-cutted nitinol stent compatible with a 10F sheath and a 0.035-inch guide wire available in eleven diameters (16–34 mm) and six lengths (30–100 mm).

**Fig. 1:**
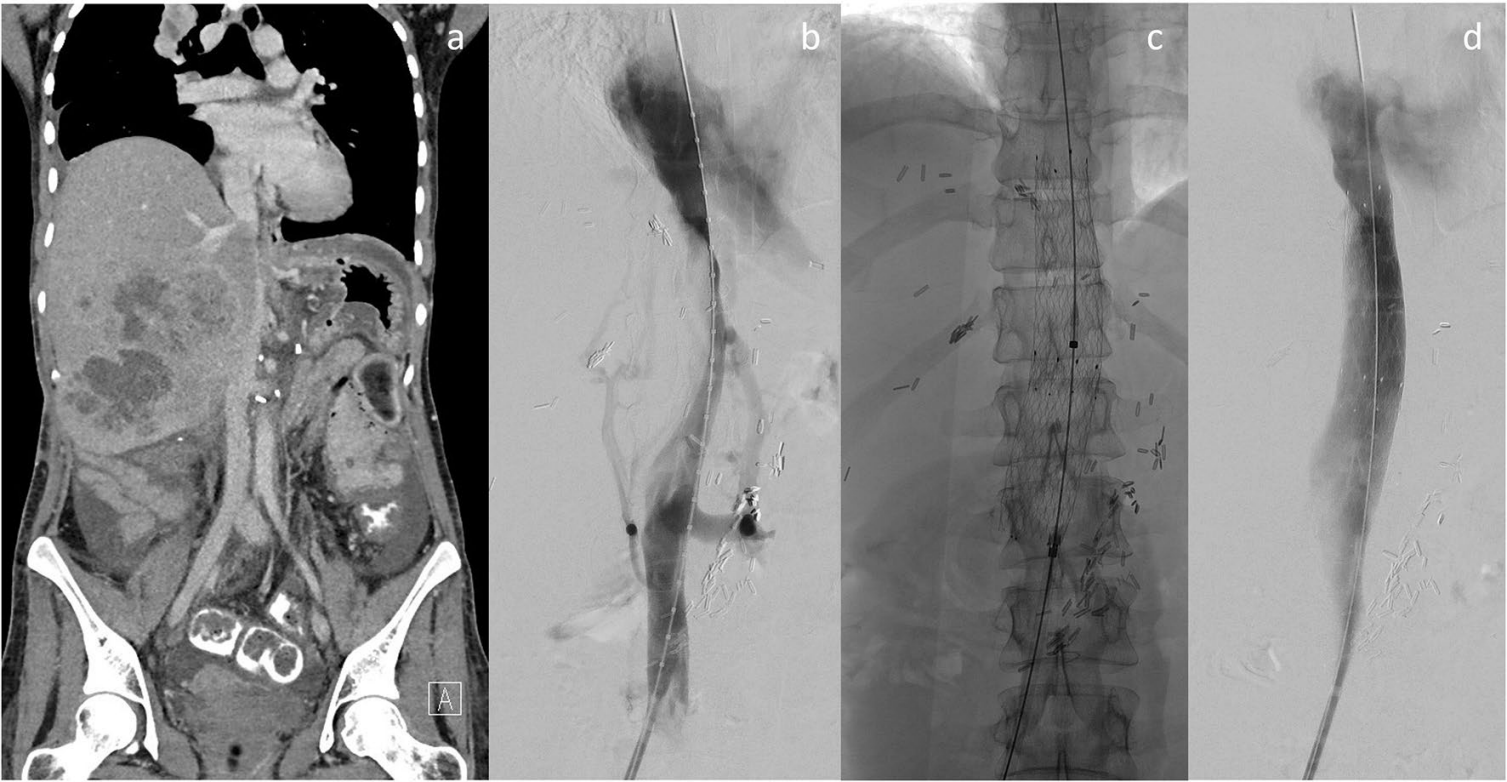
25-year-old female patient with hepatic metastasis of a retroperitoneal leiomyosarcoma. **a** Preinterventionally performed coronal contrast-enhanced computed tomography demonstrates high-grade stenosis of the IVC in the intrahepatic segment due to the intrahepatic tumor masses. **b** Corresponding cavography depicts the extent of the central venous obstruction and collateralization. **c** Fluoroscopic approval of the successful deployment of the Sinus-XL stent within in the malignant stenosis. **d** Cavography of the IVC after stent deployment demonstrates restored patency with reduced blood flow via collateral vessels

**Fig. 2:**
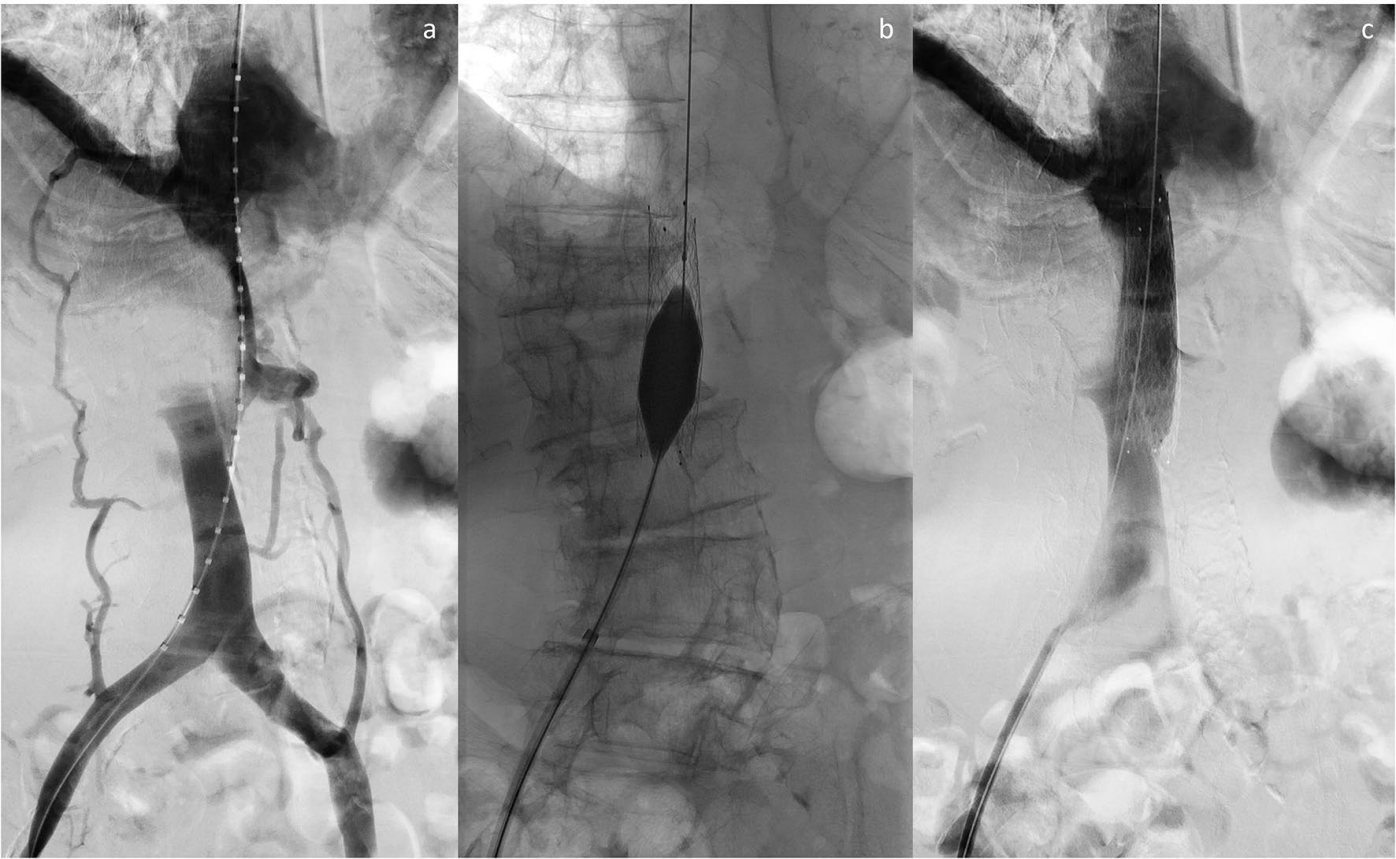
92-year-old male patient with hepatic metastasis of gastric cancer. **a** Inferior cavography depicts high-grade compression of the intrahepatic IVC with development of collateral vessels. **b** Balloon post-dilatation after deployment of the nitinol stent within the malignant stenosis. **c** Final cavography demonstrates significant improvement of the venous backflow and absence of venous collateral blood flow

After cannulation of the obstructed IVC segment, the stent system was introduced and deployed under fluoroscopic control. All patients had a bolus of 5000 units of unfractionated heparin intraprocedurally. Balloon predilatation was performed in four cases using different types of undersized balloons (Atlas®, Bard, Tempe, AZ; Zelos®, Optimed, Ettlingen, Germany; XXL® Boston Scientific, Maple Grove, MN). In nine cases more than one stent was required and implanted in an overlapping technique. In six cases (27.3%), cavography demonstrated relevant residual stenosis of the treated vessel segment, necessitating additional balloon dilatation. For post-dilatation we used undersized balloons (Atlas®, Bard, Tempe, AZ; Zelos®, Optimed, Ettlingen, Germany; Armada®, Abbott Vascular, Santa Clara, CA). A final cavogram was performed to proof sufficient stent expansion and recanalization of the stenotic IVC segment. Sheaths were removed and access site was closed by manual compression (Figs. 1, 2).

In nine patients, venous blood pressure measurements were performed during the intervention. At the discretion of the interventional radiologist, pressure gradients were measured across the stenotic segment before and after stent placement. Postinterventionally, the patients received a therapeutic PTT-adapted dose of low-molecular-weight heparin for 7 days, followed by conversion to long-term anticoagulation with enoxaparin or aspirin.

### Endpoint definition and data gathering

Two authors reviewed the patients’ medical and radiologic records to gather information on the technical and clinical success of the interventions. As far as possible, missing data were added by telephone interviews with patients and referring physicians. Technical success, clinical success and safety were defined as primary endpoints.

Technical success was designated as successful deployment of the stent within the obstructive lesion accompanied by significant improvement of blood flow in the stenotic IVC segment and by reduced blood flow via collateral vessels.

Clinical success was determined as the improvement of the patient’s symptoms. The introduction of a scoring system allowed the quantification of clinical success assessing the main manifestations, i.e. edema, anasarca and ascites before and after the procedure. To this end, we modified previously published grading systems [6, 10, 11].

Lower body edema was graded as follows: 0 for absence of edema, 1 point for mild edema, 2 points for moderate edematous swelling, 3 points for severe swelling impeding the palpation of foot pulses without pain and immobility and 4 points for massive edematous swelling causing pain and immobility. The edema distribution was scored, ranging from 0.5 points for swelling of the feet to 2 points for the genitals being involved. The presence of complicating factors, for example ulcerations, were graded with an additional point resulting in a maximum score of 7 points. Progress of the edema severity during the follow-up period was rated with another 0.5 points, whereas improvement was assessed with minus 0.5 points. The rating of lower body edema was based on the patients’ clinical reports at three time points during the follow-up period.

Ascites was scored by means of a four-point-scale: 0 for absence of ascites up to 3 indicating the necessity of paracentesis. Cross-sectional imaging and the patients’ data including paracentesis reports allowed the assessment of ascites. After the procedure, reassessment was performed at two time points during the follow-up period. During the follow-up period, a slight progression or reduction was rated by addition or subtraction of 0.5 points, a severe progression or reduction of ascites by addition or subtraction of 1 point.

Scoring of anasarca was based on cross-sectional studies exclusively. Rating was as follows: 0 point for the absence of anasarca, 1 point for slight anasarca and 2 points for severe anasarca. Anasarca scoring during the follow-up was executed analogously to the scoring of ascites.

Considering the procedural safety, complications were classified according to the reporting standards of the Society of Interventional Radiology [12].

Secondary endpoints were defined as procedural parameters, including the number and size of the implanted stents, procedure duration, fluoroscopy time, and radiation exposure in terms of the dose area product. In nine patients, pressure gradient measurements were performed before and after the stenting and the results were also added to the secondary endpoints. Furthermore, lengths of the VCI obstruction were measured based on the intraprocedural cavography as well as cross-sectional imaging.

As another secondary endpoint, we analyzed luminal expansion and stent patency. A residual stenosis of less than 50% on cross-sectional imaging is considered as luminal expansion. Patency was defined as preserved stent perfusion documented by contrast-enhanced imaging or duplex ultrasound. Luminal expansion measurements were available in 15 cases, while contrast enhanced images enabling the assessment of patency were only available in 14 cases. Assisted primary functional patency was defined as patency subsequent to another interventional treatment.

### Statistical analysis

Descriptive data were presented as means ± standard deviation (SD) for normally distributed variables or medians with ranges for non-normalized variables, if appropriate; categorical data were expressed as counts and percentages with *n* (%). With regard to assessment of normality, the Anderson–Darling test was used rejecting the hypothesis of normality when the *p* value is less or equal to 0.05. The Wilcoxon test and the Mann–Whitney U test were used for comparison of the pre- and postinterventional data or described subgroups. Kaplan–Meier analysis including the log rank test was used to analyse patients´ survival and stent patency rates.

Correlation analysis of ordinal and metrical data were performed with the test according to Spearman for non-normalized variables. For all evaluations, a *p* value less than 0.05 was considered to indicate significant differences. Statistical analysis and the evaluation of the data were performed with a specialized computer algorithm (Microsoft Excel V1908 and RStudio 1.2.5033).

### Follow-up

Clinical examination was performed in those patients who returned for routine follow-up control in our outpatient clinic. All patients were advised to immediately contact the outpatient clinic at onset of new or worsening symptoms. Imaging follow-up was conducted in the context of re-evaluation of the underlying malignant disease. Median clinical follow-up was 65 days (range 1–790 days) and 10 days (range 0–185) prior to the patients’ decease. The average time between procedure and the last cross-sectional imaging follow-up amounted to 66 days (range 1–775 days) or 35 days (range 1–179 days) between the last imaging follow-up and the patient’s decease.

## Results

### Technical success and procedural data

In all procedures, correct stent placement led to anatomical improvement of the outflow situation with marked reduction of collateral vessel flow. The technical success rate was 100% (21/21). In one case (4.8%), a reintervention was necessary three days after the procedure. This patient presented persisting edematous swelling of the lower extremities. Duplex ultrasound revealed absence of perfusion in the IVC due to significant stent compromise at the proximal end. Subsequently, the patient was transferred to the angiography suite where fluoroscopy proved the Sinus-XL stent to be subtotally compressed. Since the stent lumen could not be restored by balloon angioplasty, the stent was prolongated by a second Sinus-XL stent. This measure provided sufficient lumen restoration. As a result of this reintervention, the patient experienced a distinct clinical improvement and complete regression of the lower body edema until he deceased 389 days after the primary procedure. 

Including the case of reintervention, a total of 37 Sinus XL stents were implanted (median 1; range 1–4 stents*).* Balloon dilatation before stent deployment was performed in 18.2% of the procedures (4/22). Pressure gradient data before and after the revascularization were available in nine of the included cases (42.9%). In these cases, mean pressure gradients across the stenotic segment decreased with statistical significance from 12.7 ± 6.5 to 3.4 ± 3.0 mmHg after the stent implantation, resulting in a mean pressure gradient reduction of 64% (mean reduction 9.3 mmHg; *p* = 0.008). In eight cases, the stents covered the renal vein ostia without causing a deterioration of the renal function. Procedural data are reflected in Table 2.

**Table 2:** Procedural data

Number of stents per patient	*n*	%
1	13	59.1
2	5	22.7
3	2	9.1
4	2	9.1
Procedure time (min)	Median	Range
	22.5	7–58
Radiation exposure data	Median	Range
Dose area product (Gy*cm^2^)	46.1	10.3–805.4
Fluoroscopy time (s)	5.4	2.2–20.3
Iodinated contrast agent (ml)	Median	Range
	80	40–200
VCI obstruction length (mm)	Median	Range
	77	24–182
Stent diameter (mm)	Median	Range
	22	16–34
Stent length (mm)	Median	Range
	60	40–100

### Stent patency

Six patients died from progressive malignant disease within four weeks after the procedure. Therefore, assessment of luminal expansion was feasible in 15 patients (71.4%). Since only non-enhanced CT imaging was available in one case, we evaluated the stent patency in 14 patients. The follow-up revealed a primary stent patency of 92.9%. In one case, reintervention resulted in restoration of stent patency, leading to a primary assisted patency rate of 100% (14/14). In 46.7% of patients (7/15) we recorded stent lumen compromise of more than 50%. Information on the distribution of luminal expansion during the follow-up examinations is presented in Table 3.

**Table 3:** Luminal expansion of the Sinus-XL stent during the follow-up period (*n* = 15)

Degree of luminal expansion (%)	Number of cases (*n*)
100	3
80–100	1
70–80	1
60–70	2
50–60	1
30–40	1
20–30	2
20–10	1
< 10	3

### Complications

Major complications in terms of stent dislocation, stent migration or vascular rupture were not observed during the follow-up period. A total of three minor complications were documented (14.3%). In one case, the patient presented slight bleeding from the puncture site, which was treated successfully by manual compression (SIR complication category B). One patient complained of abdominal pain after the procedure, which was successfully treated by adjustment of the analgetic therapy (SIR complication category B). In another patient with abdominal pain subsequently to the procedure the complaints ceased without any further therapy (SIR complication category A).

### Assessment of clinical success

The included 21 patients presented several clinical symptoms before treatment. 20 patients (95.2%) suffered from edema of the lower extremities, 18 patients (85.7%) from ascites and 18 patients from anasarca (85.7%). The majority of our patients (16/21; 76.2%) suffered from a combination of all three assessed symptoms. Summarized data regarding the clinical outcome are shown in Table 4.

**Table 4:** Clinical outcome data

	*n*	Median score points	Range score points	*p* value	Clinical success (%)	Clinical success (*n*)	Median follow-up duration (d)
Edema							
Before	21	4.5	0–7				
First follow-up	17	3.5	2.5–6.5	< 0.001	82.4	14/17	2 (1–3)
Last-follow-up	21	2.5	0–6.5	< 0.001	85.7	18/21	65 (1–790)
Ascites							
Before	21	1.0	0–3				
First follow-up	19	1.0	0–3	0.172	5.3	1/19	8 (1–228)
Last follow-up	19	2.0	0–3	0.001	0	0/19	64 (1–775)
Anasarca							
Before	21	1.0	0–2				
First follow-up	14	1.0	0–2	0.125	42.9	6/14	20 (1–228)
Last follow-up	14	1.0	0–2	0.377	42.9	6/14	66 (1–775)

### Lower extremity edema

Data regarding lower extremity edema prior to the procedure were available in all 21 cases. Before the procedure, the median score for edema was 4.5 points (range 0–7 points). 66.7% of our patients (14/21) were rated with more than 4 points. Complicating conditions, leading to an additional rating point, were present in seven patients and included edematous dermatitis in four patients (19.1%), ulceration of the lower extremities in two patients (9.5%) and erysipelas in one patient (4.8%). The clinical success of the procedure with respect to symptomatic edematous swelling at the first follow-up time point was 82.4% (14/17). Complete regression of all preexisting complicating conditions were found during the follow-up period. Among 20 patients with lower extremity edema prior to the procedure, eight showed complete edema regression (40.0%).

Involvement of the hepatic segment in IVC obstruction did not show significant correlation with the degree of edema score reduction (*p* = 0.691). Spearman correlation analysis resulted in a significant correlation of stenosis length and clinical improvement. Consequently, a higher score point reduction was recorded for less than 80 mm obstruction lengths (correlation = − 0.499; *p* = 0.025). We did not find a significant correlation of intraprocedural pressure gradient reduction and clinical outcome regarding lower extremity edema at the last follow-up (correlation = 0.293; *p* = 0.482).

### Ascites

Preinterventional ascites score with a median of 1 (range 0–3) points was assessed in all patients within a median of 8 days prior to the procedure (range 1–28 days). 38.1% (8/21) of the patients had a score of 2 points. In two patients, follow-up data in terms of ascites was not available. Overall, postprocedural score points based on the last follow-up examination revealed a worsening of ascites with significantly higher median score points of 2 (*p* = 0.001). Four patients received paracentesis during the follow-up period (19.1%).

### Anasarca

**Fig. 3:**
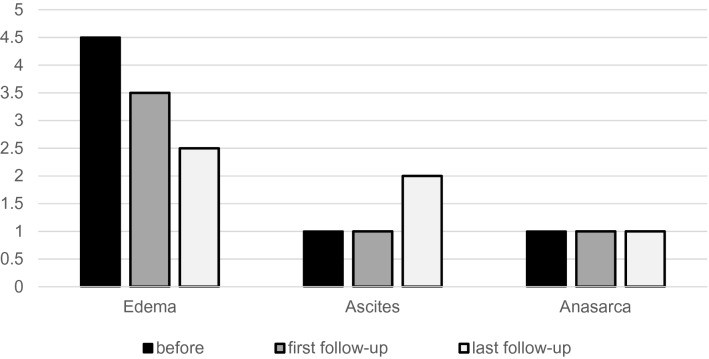
Development of score points during the study period

Evaluation of preinterventional anasarca was feasible in all included patients, resulting in a median of 1.0 point (range 0–2.0 points), with 47.6% (10/21) being rated with 2 points. Assessment of postinterventional anasarca was possible in 14 patients. Among those patients, only seven patients underwent more than one follow-up imaging, thus in 7 out of 14 patients the first and last follow-up refers to the same score. Even if a slight improvement of anasarca could be documented during the follow-up period, median score points did not change significantly. As a result, the clinical success rate concerning anasarca was 42.9% including a complete regression in 9.1% (Fig. 3).

### Survival analysis

**Fig. 4:**
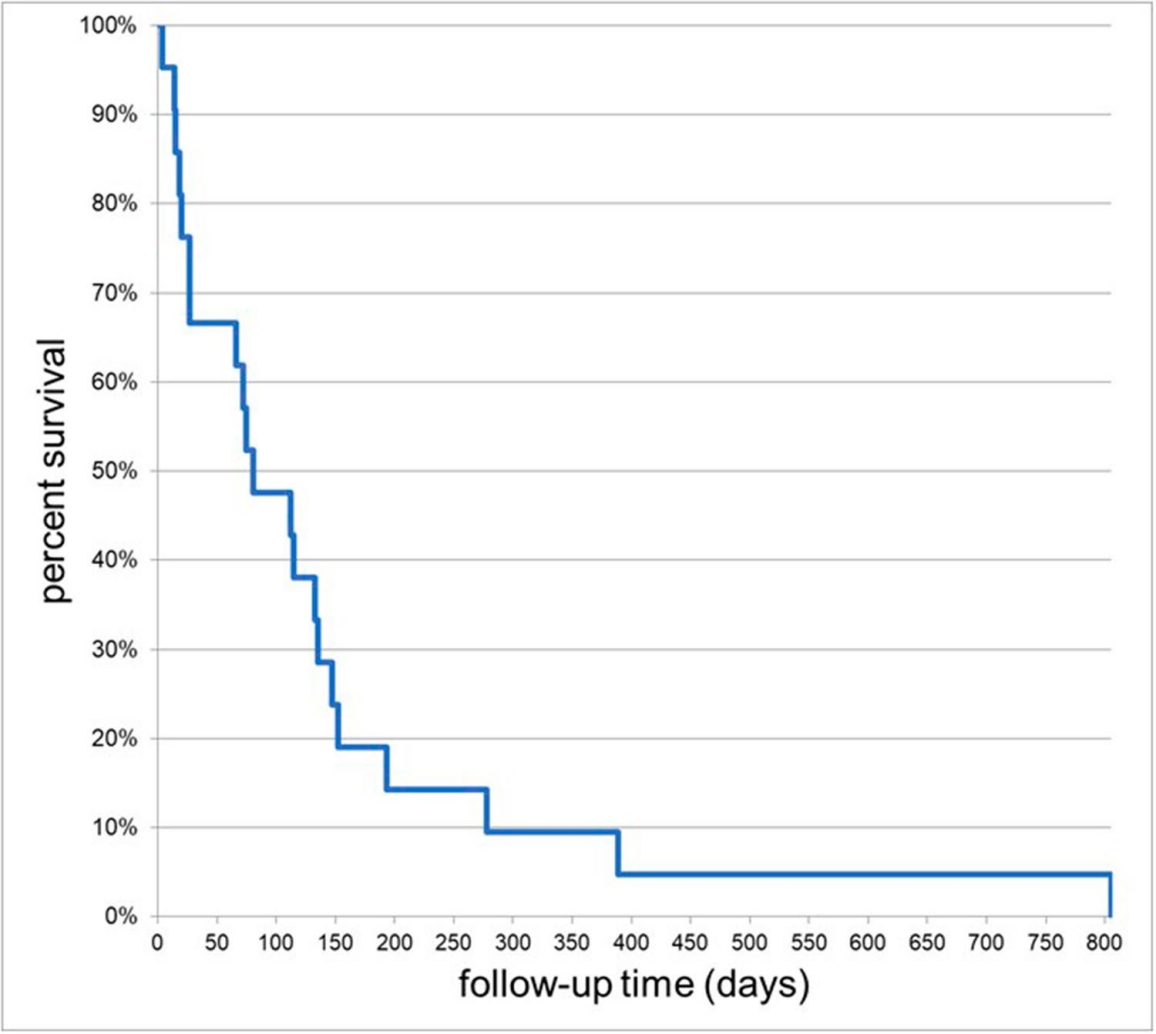
Kaplan–Meier survival curve during the follow-up. Median survival time of the patients was 81 days (4–805 days) after the procedure

During the first 30 days after the procedure, 33.3% of the treated patients (7/21) deceased. Within the follow-up period, all included patients died from the underlying advanced malignant disease (Fig. 4). Median survival time of the patients was 81 days after the procedure (range 4–805 days). Compared to the male patients, the median survival time of the female patients revealed to be 37 days shorter (median 75.0; range 4–152 days vs. median 112 days; range 14–805 days; *p* = 0.101). Patients with IVC occlusion at the level of the intrahepatic vessel segment showed shorter survival times compared to those without involvement of the intrahepatic segment (median 66.0; range 4–389 days vs. median 133.0; range 14–805 days; *p* = 0.168). Survival time revealed to be longer in patients with adjuvant oncologic treatment after IVC stenting (median 115; range 66–805 days vs. median 18; range 4–27 days; *p* = 0.247). Nevertheless, none of those trends reached statistical significance.

## Discussion

To our knowledge, to date our study is the first to be characterized by both, the utilization of the same closed-cell designed stent type throughout all the interventions, and the same experienced interventional radiologist performing all the procedures. In the field of malignant SVC obstructions, the application of the laser-cut nitinol Sinus-XL stent has already reached high levels of feasibility, safety, and technical success [13].

Patients with malignant IVC obstruction are predominantly characterized by advanced and incurable tumor stages, which is also reflected by the noticeable short survival times found in our study collective [14]. Given this fact, studies dealing with the symptomatic treatment of these patients can only be carried out to a limited extent and commonly lack long-term outcome analysis [4]. Studies addressing IVC stenting in particular remain limited and are commonly reported in the context of heterogeneous patient cohorts including SVC obstructions as well [9, 15–17]. One major reason for this fact is that malignant IVC obstruction is a rare condition [18].

As an alternative to IVC stenting, the use of chemotherapy or radiotherapy might achieve some degree of symptom relief, but presupposes a sufficient sensitivity of the underlying malignancy towards the treatment. Taken the acuteness of severe IVC compression symptoms into account, a successful, immediate therapy response due to IVC stenting offers a promising tool to palliate these critically ill patients [19, 20]. In our study, patients with subsequent radiotherapy and/or chemotherapy following stent implantation experienced a better outcome in terms of longer survival times, which agrees with other results regarding SVC stenting, even if this trend did not reach statistical significance [20, 21]. In this vulnerable patient collective, invasive surgical resection techniques are mostly not an option owing to the high operative risk [22]. The potentially fast implementation is one of the main advantages of the described procedure. In our study collective, the average procedure time amounted to 26 min which is considerably shorter than in previously published studies. This is supposed to be a result of the operator’s experience [10].

The present study focuses on the clinical outcome. In contrast to previously published data, radiological imaging was also included in the assessment of clinical success. Furthermore, a detailed, point-based score graduation was developed to assess both, the initial assessment of clinical status, as well as the course of symptoms. Overall, our results suggest that IVC stenting effectively alleviates lower extremity edema, which is one of the major symptoms associated with IVC obstruction. On the other hand, ascites and anasarca revealed only to be slightly influenced by stent treatment. Ascites and anasarca may be caused by a variety of pathologic conditions, such as disturbed venous outflow, impaired liver function in extended hepatic metastasis, peritoneal carcinomatosis, or cardiac insufficiency [23]. Therefore, the amount of ascites or anasarca can hardly be influenced by IVC stent placement as it is evidenced by our study results. Oppositely, even if edema can also be the result of different pathologic imbalances, it is most often based on a disturbed venous outflow, especially when being localized in a circumscribed area.

We stated that the overstenting of the renal vein ostia did not lead to an elevation of creatinine levels. Thus, we suppose that the stent strut gaps enable the renal venous outflow to be sufficiently preserved. Even if renal vein ostia overstenting causes acute venous outflow blockage, we assume that the renal venous blood finds its way through collateral pathways, for example through the periureteric venous plexus and the left gonadal vein [1, 24].

Compared to previously published data, the rate of reinterventions revealed to be relatively low (4.8%) with only one case necessitating early restoration of the stent lumen. For example, Fatima et al. reported a reintervention rate of 14.3% (4/28) after stenting in chronic thrombotic IVC occlusion [25]. Furthermore, previously reported severe procedure related complications, e.g. pulmonary edema, pulmonary embolism and stent fracture, did not occur in our study [6, 11, 26]. Intraprocedural and long-term stent migration as reported in other studies were not encountered in our case series [8, 13].

In accordance with Mokry et al., who implanted Sinus-XL stents in patients with superior vena cava compression syndrome, we used an undersized balloon for postdilatation, since the high radial force of the self-expanding Sinus-XL stent requires postdilatation only at the level of the tumor stenosis maximum [13]. In our experience, the use of balloons equal or above the reference vessel diameter for postdilatation may be accompanied by stent recoiling at the level of the tumor maximum. Moreover, we hypothesize that the use of undersized balloons may prevent excessive tumor ingrowth.

It remains a matter of debate, whether covered stents should be preferred. Gwon et al. reported significant longer patency rates following covered stent placement in the treatment of SVC obstruction, thus the use of the non-covered Sinus-XL stent in the treatment of malignant IVC obstruction can be questioned [27]. However, even if the covered stenting might be an advantage in view of stent patency, the fact that a bare-metal design allows flow preservation via renal veins and collateral vessels can be considered as prevailing benefit.

The present study underlies different limitations. First, the study design is retrospective, and the number of included patients is limited. In synopsis with the patients´ critical condition, clinical and radiological follow-up examinations are inconsistent and partly incomplete. Second, the included patient collective is heterogeneous regarding the underlying malignant disease, the level of venous obstruction, and previously as well as subsequently received treatment. The lack of established criteria in the assessment of IVC symptoms represents another a general obstacle in a reliable and applicable outcome evaluation. The application of clinical scores, especially in a retrospective rating design, is subjective and thus impedes the comparison of the respective study results.

Even though malignant IVC obstruction is rare and the analyzed patient collective is small, our study offers further insights into the technical aspects and the clinical outcome of the described procedure. To gain more detailed and transferable data, a larger and multicentric study applying a consensus-reading of a standardized score rating system would be desirable. Randomization might additionally facilitate the comparison of different stent types.

Based on our data, the utilized nitinol stent gathers the following advantages. The Sinus-XL stent appears to provide a patency comparable to other stent types. Concerning luminal expansion, comparable data are not available. However, contrary to balloon expandable stents, the self-expanding, closed-cell designed Sinus-XL stent is characterized by a higher radial force delivering increased resistivity against tumor compression [28]. Further, we did not observe the foreshortening effect reported in the context of the WALLSTENT implantation [4, 13, 15, 29–31]. With respect to luminal tumor ingrowth, the nitinol Sinus-XL stent offers more resistance due to its close-meshed stent design compared to coarse-meshed designed stents, e.g. the self-expanding stainless-steel Gianturco stent. In addition, the Gianturco stent revealed only little resistance against eccentric tumor compression, which questions its suitability in the treatment of malignant IVC outflow obstruction [32, 33].

In conclusion, IVC stent implantation utilizing the Sinus-XL system is a feasible, effective and safe minimally invasive procedure, enabling significant clinical improvement of lower extremity edema in patients suffering from malignant IVC obstruction syndrome. In contrast, a relevant benefit concerning ascites and anasarca was not observed. In this vulnerable patient cohort, the minimally invasive procedure offers excellent technical success rates, prompt clinical success and a low complication rate. Therefore, it is considered suitable in a palliative care setting. Due to the limited life expectancy, the procedures´ long-term results remain insufficiently elucidated.

## Data Availability

The data that support the findings of this study are available from the corresponding author upon reasonable request.
